# Applying prospective tree-temporal scan statistics to genomic surveillance data to detect emerging SARS-CoV-2 variants and salmonellosis clusters in New York City

**DOI:** 10.1093/ije/dyaf032

**Published:** 2025-04-10

**Authors:** Sharon K Greene, Julia Latash, Eric R Peterson, Alison Levin-Rector, Elizabeth Luoma, Jade C Wang, Kevin Bernard, Aaron Olsen, Lan Li, HaeNa Waechter, Aria Mattias, Rebecca Rohrer, Martin Kulldorff

**Affiliations:** Division of Disease Control, New York City Department of Health and Mental Hygiene, Long Island City, NY, United States; Division of Disease Control, New York City Department of Health and Mental Hygiene, Long Island City, NY, United States; Division of Disease Control, New York City Department of Health and Mental Hygiene, Long Island City, NY, United States; Division of Disease Control, New York City Department of Health and Mental Hygiene, Long Island City, NY, United States; Division of Disease Control, New York City Department of Health and Mental Hygiene, Long Island City, NY, United States; Division of Disease Control, New York City Department of Health and Mental Hygiene, Long Island City, NY, United States; Division of Disease Control, New York City Department of Health and Mental Hygiene, Long Island City, NY, United States; Division of Disease Control, New York City Department of Health and Mental Hygiene, Long Island City, NY, United States; Division of Disease Control, New York City Department of Health and Mental Hygiene, Long Island City, NY, United States; Division of Disease Control, New York City Department of Health and Mental Hygiene, Long Island City, NY, United States; Division of Disease Control, New York City Department of Health and Mental Hygiene, Long Island City, NY, United States; Division of Disease Control, New York City Department of Health and Mental Hygiene, Long Island City, NY, United States; Independent Biostatistician, Ashford, CT, United States

**Keywords:** food-borne diseases, infectious diseases, SARS-CoV-2, *Salmonella*, surveillance, whole-genome sequencing

## Abstract

**Background:**

The detection of communicable disease clusters in genomic surveillance data typically involves the application of rule-based signaling criteria, which can be arbitrary. In contrast, scan statistics that are used for spatiotemporal cluster detection can flexibly scan in calendar time, and scan statistics that are used for pharmacovigilance can flexibly scan along hierarchical tree structures that are based on diagnosis codes.

**Methods:**

New York City (NYC) Health Department staff applied tree-temporal scan statistics prospectively to genomic surveillance data with a hierarchical nomenclature for COVID-19 and salmonellosis cases that were diagnosed among NYC residents. We searched weekly for recent case increases at any granularity, from large phylogenetic branches to small groups of indistinguishable isolates. Using free and open-source TreeScan software, we looked for emerging SARS-CoV-2 variants based on Pango lineages during August 2021–November 2023 and emerging clusters of *Salmonella* isolates based on allele codes during November 2022–November 2023.

**Results:**

The SARS-CoV-2 Omicron subvariant EG.5.1 first signaled as locally emerging on 22 June 2023, 7 weeks before the World Health Organization designated it as a variant of interest. During 1 year of salmonellosis analyses, TreeScan detected 15 credible clusters that were worth investigating for common exposures and two data-quality issues for correction.

**Conclusion:**

A challenge was the maintenance of timely and specific lineage assignments, and a limitation was that genetic distances between tree nodes were not considered. By automatically sifting through genomic data and generating ranked shortlists of nodes with statistically unusual recent case increases, TreeScan assisted in detecting emerging variants and clusters of communicable diseases and in prioritizing them for investigation.

Key Messages

- Current practices for the detection of emerging disease clusters and variants in genomic surveillance data often rely on arbitrary criteria and time-consuming, manual laboratory data review.
- Tree-temporal scan statistics can detect emerging clusters by flexibly scanning prospectively in calendar time while also flexibly scanning along a tree structure, such as for genetic relatedness.
- We detected credible disease clusters for investigation and data-quality problems for correction, which helped health department officials to focus investigative resources.

Whole-genome sequencing (WGS) data are increasingly used by public health officials for communicable disease surveillance and cluster detection [1]. For example, SARS-CoV-2 variant surveillance allows officials to monitor the effects of new variants on COVID-19 disease severity, transmission, diagnostics, therapeutics, and immunity from prior infections and vaccinations [2]. In the USA, variant data have guided decisions around COVID-19 vaccine composition and the revocation of emergency-use authorizations for monoclonal antibody therapies with decreased clinical efficacy [3]. Such data have been used in New York City (NYC) to summarize epidemiologic characteristics of newly emerging variants [4], assess illness severity [5], and elucidate community transmission patterns [6]. Timely knowledge of emerging variants with increased transmissibility or immune escape can prompt actions to limit spread. Such actions are particularly important in congregate settings and for populations who are at increased risk of severe illness, such as people who are older or living with comorbid conditions [7].

When many SARS-CoV-2 variants and recombinants co-circulate, a key challenge is deciding in near real time which ones to closely monitor over which time increments. Bioinformatic methods and phylodynamic models can be used to estimate variant-specific growth rates and prioritize variants [8], although this can be onerous to operationalize with many co-circulating variants. In the COVID data tracker that was developed by the US Centers for Disease Control and Prevention (CDC), lineages are displayed if they either account for >1% of sequences nationally during a 2-week period or have been classified as a variant of interest or concern [2, 9].

Moreover, WGS-based subtyping is revolutionizing population-based enteric bacterial disease surveillance. When officials can quickly identify patients who are infected with genetically similar pathogens, the probability of identifying a common exposure and preventing further infections is increased [10]. The Public Health Laboratory (PHL) at the NYC Health Department performs core genome multilocus sequence typing (cgMLST) by using prescribed CDC PulseNet methods [11]. To detect *Salmonella* clusters, PHL staff compare cgMLST profiles of sequences that are stored in a local database of isolates (pathogens isolated from clinical specimens) that have been tested and sequenced at PHL. This process excludes isolates that are sequenced out-of-jurisdiction and also requires time-consuming manual input, which can lead to missed or delayed cluster detection.

Enteric disease cluster detection is typically operationalized by using static, rule-based definitions in which isolates from patients in a geographic area are grouped within fixed cutoffs of genetic relatedness and time [12, 13]. A commonly used working cluster definition is ≥3 *Salmonella* clinical isolates within a 60-day window within 10 alleles, of which ≥2 cases are within ≤5 alleles [11, 14]. Such rules, which can be arbitrary, vary across pathogens according to genetic diversity, ecology, and prevalence [15]. Existing cluster detection tools [16–18] do not also analyse the extent to which cases were spread out versus were concentrated in time, despite the importance of temporal clustering for cluster detection and investigation.

In contrast, space–time scan statistics search flexibly in both space and time [19, 20]. Several public health institutions previously used a rule-based aberration detection method (the historical limits method) [21]; CDC discontinued this approach in 2020 [22]. In 2014, the NYC Health Department transitioned to using scan statistics to quickly detect unusual clusters of any geographical size or duration for many reportable communicable diseases [23]. We wish to similarly search WGS data in a flexible manner in time. However, rather than flexibly searching for increases in geographical location and size, we wish to be flexible in the location of the patients’ WGS isolates on a phylogenetic tree and the granularity of nodes on that tree.

Tree-temporal scan statistics [24, 25] are used by CDC, the US Food and Drug Administration, and academic scientists to detect and evaluate unanticipated adverse reactions to pharmaceutical drugs and vaccines [26–28]. In this pharmacovigilance context, potential adverse events can be classified in a tree structure based on the International Classification of Diseases, Tenth Revision (ICD-10) diagnosis codes. The codes are grouped hierarchically, reflecting general or specific disease conditions that affect different body systems, with related diagnoses located on the same tree branch. Unusual increases in diagnoses at any level of specificity can be detected in sequential analyses, at any length of time after vaccine or drug administration.

Herein, we marry ideas of flexibly scanning prospectively in calendar time (as for spatiotemporal cluster detection) with flexibly scanning along a hierarchical tree structure (as is conducted for pharmacovigilance). We thereby establish an “innovation at the edge” of infectious disease epidemiology and pharmacoepidemiology [29]. We describe the real-time application of prospective tree-temporal scan statistics by the NYC Health Department. We selected SARS-CoV-2 and *Salmonella* because of their substantial disease burdens and availability of genomic surveillance data with a hierarchical nomenclature, with the potential to guide local public health actions.

## Methods

### Genomic surveillance data

#### SARS-CoV-2

PHL and other laboratories perform WGS on a portion of specimens from confirmed COVID-19 cases [30] that are diagnosed among NYC residents, as previously described [4, 31]. Weekly, starting on 12 August 2021, we determined the counts of each lineage assignment during a rolling 12-week period, ending on the most recent specimen collection date (Table 1). Pango lineages, which represent the dynamic nomenclature applied to genetically distinct SARS-CoV-2 lineages [32], were assigned by using the pangolin software tool [33]. Initially, we used the PangoLEARN machine-learning model to assign a lineage name to each WGS result [33]. To improve lineage assignment stability, on 16 December 2021, we switched to the UShER method for placing new genome sequences onto a phylogeny [34].

**Table 1.** Specifications for analyses using tree-temporal scan statistics applied prospectively to genomic surveillance data among NYC residents

Feature	SARS-CoV-2	*Salmonella*	Notes
Genomic data resolution	Pango lineages	Allele codes	The “nodes” in our genomic surveillance trees represent SARS-CoV-2 variants or *Salmonella* allele codes
Temporal element	Specimen collection date	Specimen collection date (or upload date, in sensitivity analyses)	The specimen collection date is the most epidemiologically relevant date, representing when patients sought care. For *Salmonella*, to accommodate delays between specimen collection and allele code assignment, we also conducted sensitivity analyses, in which the temporal element was the date uploaded to the System for Enteric Disease Response, Investigation, and Coordination (SEDRIC)
Time precision	Day		We used data at daily resolution (as opposed to aggregating by week or month) to improve precision in cluster start dates
Study period	12-week period ending on the most recent specimen collection date	1-year period ending on the most recent specimen collection (or upload) date	For SARS-CoV-2, due to rapid variant turnover, we used a short study period that was three times as long as the maximum temporal cluster size (see below). For *Salmonella*, we used the standard study period of 1 year [35]
Only allow data on leaves of tree	No		For genomic surveillance data, valid patient results could be anywhere on the tree, not only at the most specific nodes
Allow multiple parents for the same node	Yes	No	We assigned multiple parents for recombinant SARS-CoV-2 lineages, effective in January 2024. For *Salmonella*, each node had only one parent
Type of scan	Tree and time		We scanned for increases in cases at any node or group of related nodes and over any recent time period
Conditional analysis	Node and time		We conditioned on time to adjust nonparametrically for any citywide purely temporal patterns, such as data-reporting lags or increasing or decreasing trends. We also conditioned on node to account for whether cases historically had been common or rare at each node during the baseline period. This is because we were interested in detecting newly emerging nodes, not nodes that were also common during the baseline period
Scan for branches with:	High rates		We wished to detect clusters as they emerged rather than declined
Maximum temporal size	28 days	90 days	For SARS-CoV-2, we searched for increases in variants during the most recent 14, 15, 16, …, 27, or 28 days to balance recency and persistence. For *Salmonella*, we searched for allele codes with increases during the most recent 1, 2, 3, …, 89, or 90 days to encompass the standard 60 days in the rule-based *Salmonella* definition, plus an additional 30 days to accommodate data lags
Minimum temporal size	14 days	1 day	
Prospective evaluation	Yes		Prospective analyses were used to search for emerging clusters rather than historical clusters by only considering temporal windows reaching up to the study period end date
Perform node by day-of-week adjustment	No		Sequencing results were unlikely to vary by the day of the week on which the specimen was collected
Inference method	Sequential Monte Carlo		We used a sequential method with an early termination cutoff, which allowed runs to terminate early if there were no unusual clusters
Monte Carlo replications	999 999	99 999	To slightly improve performance, we used more than the standard 999 Monte Carlo replications, as allowed based on computing time, which is determined by the number of tree nodes and time intervals
Prospective analysis frequency	Weekly		We performed analyses weekly (as opposed to daily) to match the frequency with which input data were refreshed
Minimum number of cases	2		We retained the default minimum so as not to miss any emerging clusters
Signal definition	Recurrence interval (RI) ≥ 365 days	RI ≥ 100 days	We considered RI 100 to <365 days as a weak cluster, RI 365 days to <5 years as a moderate cluster, RI 5 to <100 years as a strong cluster, and RI ≥100 years as a very strong cluster [35]

Occasionally, as with XBB.1.5 and then XBB.1.16, a variant newly emerged during the rolling 12-week study period and quickly became the primary signaling node. In these instances, so as not to obscure more recently emerging variants, we reset the study period to begin after that variant stabilized as a percentage of sequenced cases. Once 12 weeks had elapsed since that stabilization, we returned to a rolling 12-week period. This is similar to an approach that is used to fine-tune a spatiotemporal cluster detection system when data in the temporal window and the baseline period are not comparable [35].

##### Salmonella

When a NYC resident tests positive for *Salmonella* infection, city and state laws require the laboratory to report the result and submit the patient’s isolate to PHL or the New York State Department of Health [36, 37]. These laboratories conduct WGS on the isolates. WGS data (including serotype and cgMLST allele calls) and patient demographic data are uploaded to CDC PulseNet, where allele codes are assigned at the national level [11]. Allele codes are then populated in CDC’s System for Enteric Disease Response, Investigation, and Coordination (SEDRIC). In parallel, graduate student interns at the NYC Health Department attempt to interview all NYC residents with salmonellosis as soon as is feasible after the initial report to collect possible exposure information.

Weekly, starting on 16 November 2022, we downloaded from SEDRIC the allele codes for salmonellosis (typhoidal and non-typhoidal) for New York State residents, as additional parsing of patient addresses was necessary to restrict to NYC residents. We determined counts of each *Salmonella* allele code among NYC residents during a rolling 365-day period, ending on the most recent specimen collection date (Table 1).

#### Health equity

The population benefits of genomic surveillance might be inequitably distributed if particular groups are underrepresented in WGS results [38]. Underrepresentation might be a consequence of inequitable access to healthcare and laboratory testing, and, for SARS-CoV-2 infections, nonrandom sampling practices for sequencing [31]. We assessed WGS result availability for confirmed and probable cases [30] of COVID-19 and salmonellosis among NYC residents who were diagnosed during a 2-year period that ended in October 2023. We stratified by patient-level race or ethnicity and by the Index of Concentration at the Extremes—an area-based measure of economic and racial or ethnic segregation [39].

### Hierarchical tree files

#### SARS-CoV-2

Pango lineage notes were used to determine parent–child relationships for all detected SARS-CoV-2 variants [33, 40]. For example, for the analysis that was conducted on 17 August 2023, all detected variants were descended from B.1. Thus, B.1 was designated as the tree root, which progressively branched into increasingly specific lineages, including the Omicron variant (i.e. B.1.1.529), culminating in more specific nodes, such as the Omicron subvariant EG.5.1.1. For recombinant lineages (e.g. XBB), in January 2024, we switched to assigning multiple parents, but in the earlier analyses that are presented here, we assigned the most recent common ancestor as the parent (Table 2).

**Table 2.** Example hierarchical nomenclature for SARS-CoV-2 variants assigned to Pango lineages, showing tree levels

Level	Node (Pango lineage)	Note
1	B.1	
2	B.1.1	
3	B.1.1.529	Omicron
4	BA.2	Alias of B.1.1.529.2
5	XBB	Recombinant lineage of BJ.1 (alias of B.1.1.529.2.10.1.1) and BM.1.1.1 (alias of B.1.1.529.2.75.3.1.1.1)
6	XBB.1	
7	XBB.1.9	
8	XBB.1.9.2	
9	EG.5	Alias of XBB.1.9.2.5
10	EG.5.1	
11	EG.5.1.1	

##### Salmonella

We designated ‘SAL’ as the tree root and each *Salmonella* serotype (e.g. Typhi, Enteritidis, Kottbus) as the second tree level. We appended the allele code, which can be up to six digits, to the serotype. Whereas laboratory scientists typically compare isolates manually by using allele ranges, we used allele codes because of the standardized hierarchical nomenclature. Isolates with more allele code digits in common have a lower number of allele differences (Table 3).

**Table 3.** Example hierarchical nomenclature for *Salmonella* isolates assigned a serotype and allele code

Level	Node (serotype, allele code)	Maximum expected allele difference for isolates matching at specified allele code digit
1	SAL	Not applicable
2	SAL.Kottbus	Not applicable
3	SAL.Kottbus.6185	80
4	SAL.Kottbus.6185.1	28
5	SAL.Kottbus.6185.1.1	15
6	SAL.Kottbus.6185.1.1.2	7
7	SAL.Kottbus.6185.1.1.2.1	4
8	SAL.Kottbus.6185.1.1.2.1.1	0^a^

a Isolates matching at the sixth digit of the allele code are 0 alleles apart, i.e. indistinguishable by core genome multilocus sequence typing.

### Prospective tree-temporal scan statistic

We conducted prospective analyses by using tree-temporal scan statistics [24, 41] (Table 1) in the free and open-source TreeScan^TM^ software (Martin Kulldorff and Information Management Services, Inc., Calverton, Maryland). We searched for unusual increases that emerged over any recent time period at any node, from large phylogenetic branches to small groups of genetically indistinguishable isolates. Under the null hypothesis, for each variant or group of related variants, its proportion among all variants is constant over time. Under the alternative hypothesis, for one variant or group of related variants, its proportion among all variants is higher during some recent period. Mathematical formulae for calculating the expected number of cases, excess cases, and relative risk are available in the TreeScan user guide [41].

For each node (candidate cluster), a likelihood ratio-based test statistic is calculated when the observed number of cases during the time window at the node exceeds the expected number. The candidate cluster with the maximum likelihood ratio test statistic is the cluster that is least likely to be due to chance under the null hypothesis of no node-by-time interaction, after adjusting for purely temporal variation and total node counts during the study period. For example, if a node has 5.4% of cases during the baseline period (i.e. prior to the cluster period) and there are 100 total cases with WGS results during the cluster period, then the expected number of cases during the cluster period at that node is 5.4.

Monte Carlo hypothesis testing is used to assess the statistical strength of clusters, controlling for the multiplicity of overlapping nodes and time windows evaluated. To create simulated datasets under the null hypothesis, case dates are shuffled and randomly assigned to the original nodes. The maximum likelihood ratio test statistic for each simulated dataset is calculated in the same way as for the observed dataset.

The maximum likelihood ratio for the observed dataset is ranked among those from the simulated runs under the null hypothesis, and a *P*-value is derived from this ranking as *P* = rank/(999 999 + 1) for the SARS-CoV-2 analyses [41]. For prospective analyses, a recurrence interval (RI) is calculated as the reciprocal of the *P*-value. For a weekly analysis frequency, this is further divided by 52 for the number of analyses per year. The RI represents the duration of weekly surveillance required for the expected number of clusters that are at least as unusual as the observed cluster to be equal to 1 by chance [42]. For example, when the null hypothesis of no clusters is true, then, during a 1-year period, the expected number of clusters with RI ≥ 365 days is 1. Supplementary Appendix S1 provides cluster-reporting details.

### Performance assessment

#### SARS-CoV-2

In the absence of national guidance for ways in which jurisdictions should select variants to monitor locally, we compiled illustrative examples of successes and challenges in using TreeScan results to focus attention on emerging variants during weekly analyses that were conducted during August 2021–November 2023.

##### Salmonella

We characterized clusters that were prospectively detected by using TreeScan during the first year of weekly analyses, from 16 November 2022 to 8 November 2023. We considered clusters to be “solved” if investigators identified a common food source, animal exposure, exposure site, or travel history that likely explained the association among cluster patients. Supplementary Appendix S2 provides further details about cluster definitions, cluster prioritization, and consideration of typhoidal clusters.

## Results

### Completeness and representativeness

Among NYC residents who were diagnosed during November 2021–October 2023, WGS was conducted for 7% of COVID-19 cases (151 944 of 2 266 600) and for 62% of non-typhoidal salmonellosis cases (1679 of 2722; Supplementary Table S1). Of 1068 salmonellosis cases with no allele code, 937 (88%) were probable cases with only a positive culture-independent diagnostic test result, 106 (10%) were culture-positive but had no isolate available for WGS, 5 (<1%) underwent WGS but failed quality control, and 20 (2%) were unique sequences that CDC’s naming algorithm could not match to an existing allele code. The median lag from specimen collection to allele code assignment was 22 days (interquartile range: 20–28 days). Of 1654 salmonellosis cases with an allele code assigned, 1322 (80%) had a fully or partially completed interview; interviews are necessary to collect information for identifying common exposures among cluster patients.

Patient demographic characteristics were similarly distributed between reported cases overall and the subset with WGS results. Distributions were within ±2.5% for every stratum of race or ethnicity and the Index of Concentration at the Extremes (Supplementary Table S1). Although substantial proportions of patients lacked WGS results, there was no evidence of systematic underrepresentation during this period.

### SARS-CoV-2 illustrative examples

#### Rapid detection of a locally emerging variant

**Figure 1.**
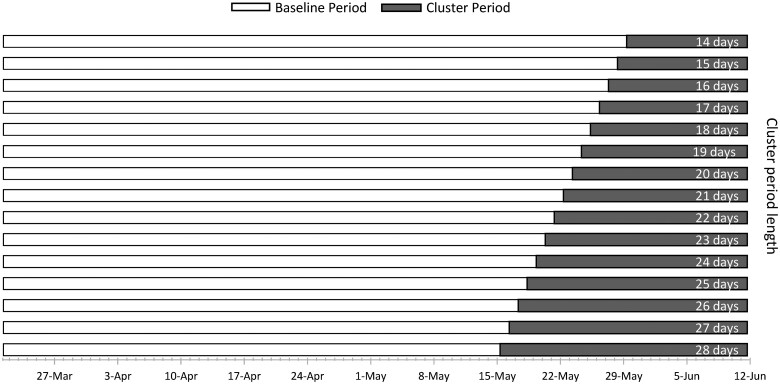
Example of prospective temporal scan windows for a 12-week study period at daily resolution ending on 12 June 2023, with 14-day minimum and 28-day maximum cluster periods. In prospective analyses, only cluster periods extending to the study period end date are evaluated. The baseline period, which is used for comparison, is prior to each cluster period.

**Figure 2.**
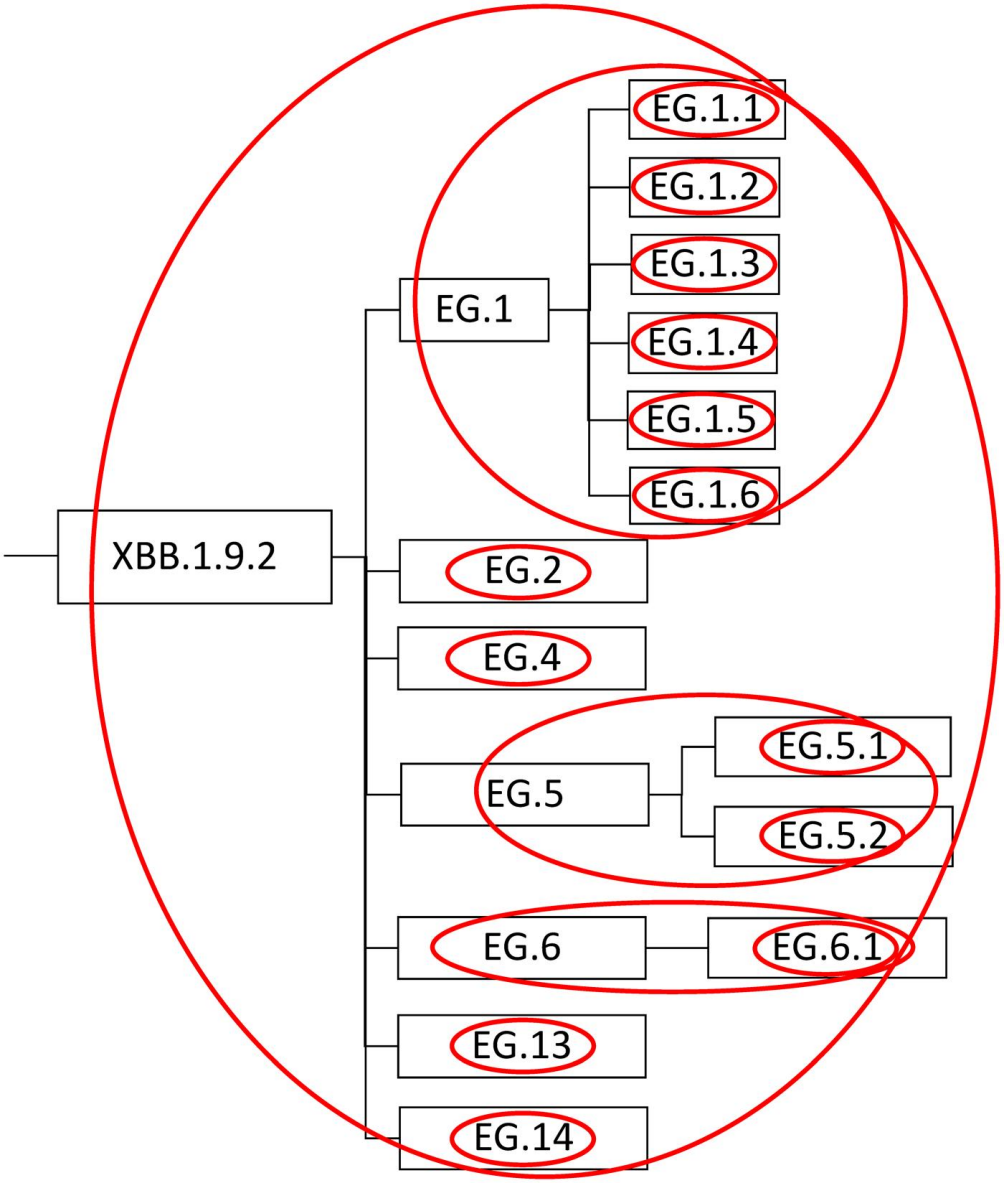
Example of scanning windows for a SARS-CoV-2 genomic surveillance tree excerpt, showing XBB.1.9.2 and its subvariants as detected among NYC residents as of 12 June 2023. Each detected variant is a node that is connected to its parent and any children. Ovals indicate the complete set of scanning windows within this excerpt, evaluating each node together with its descendants.

The analysis that was performed on 22 June 2023 searched for variants that were emerging during the 14, 15, 16, …, 27, or 28-day period that ended on 12 June 2023 (Figure 1), across 200 nodes (excerpted in Figure 2). This analysis, with a computer running time of 4 minutes and 15 seconds, identified six SARS-CoV-2 variants emerging among NYC residents (Table 4). Three of the six nodes were the same as in the prior week’s analysis (Supplementary Table S2), including persistent, strong signals for XBB.1.16, XBB.2.3, and their subvariants. Of the newly signaling nodes, EG.5.1 (RI = 35 years) first signaled more strongly than its grandparent (XBB.1.9.2, Figure 2), with 11 specimens collected during 17 May–12 June 2023. EG.5 or EG.5.1 continued to signal for 13 consecutive weekly analyses, from 22 June to 14 September 2023, after which more specific subvariants (e.g. EG.5.1.6) began to signal more strongly.

**Table 4.** Analysis conducted on 22 June 2023 to apply prospective tree-temporal scan statistics to detect emerging SARS-CoV-2 variants in specimens that were collected among NYC residents during the 14- to 28-day period that ended on 12 June 2023

Variant	No. of cases with specimens collected during 12-week study period, 21 March–12 June 2023	Cluster start date, ending on 12 June 2023	No. of cases with specimens collected during cluster window	No. of expected cases	Relative risk	No. of excess cases	Test statistic	RI (years)^b^	No. of consecutive weeks signaling	Percentage of sequenced cases with specimens collected in week ending 12 June 2023
XBB.1.16^a^	213	16 May	107	52.1	3.7	78.0	22.8	19 231	11	28
XBB.2.3^a^	83	18 May	41	17.5	3.9	30.5	11.5	4808	3	17
XBB.1.24.1	5	30 May	5	0.4	∞	5.0	8.2	55	1	<1
EG.5.1^c^	11	17 May	11	2.5	∞	11.0	7.9	35	1	6
XBB.1.5.68	8	19 May	8	1.6	∞	8.0	6.6	5	1	<1
XBB.1.5.16^a^	13	16 May	11	3.2	17.3	10.4	5.8	2	2	<1

a Subvariants included.

b Nodes with RI ≥ 1 year were included. The maximum possible RI for this analysis was 19 231 years. When using 999 999 Monte Carlo replications, the smallest possible *P*-value is 1/999 999 = 0.000001. With a weekly prospective analysis frequency, the maximum RI was thus (1/0.000001)/52 analyses per year = 19 231 years.

c Alias of XBB.1.9.2.5.1.

**Figure 3.**
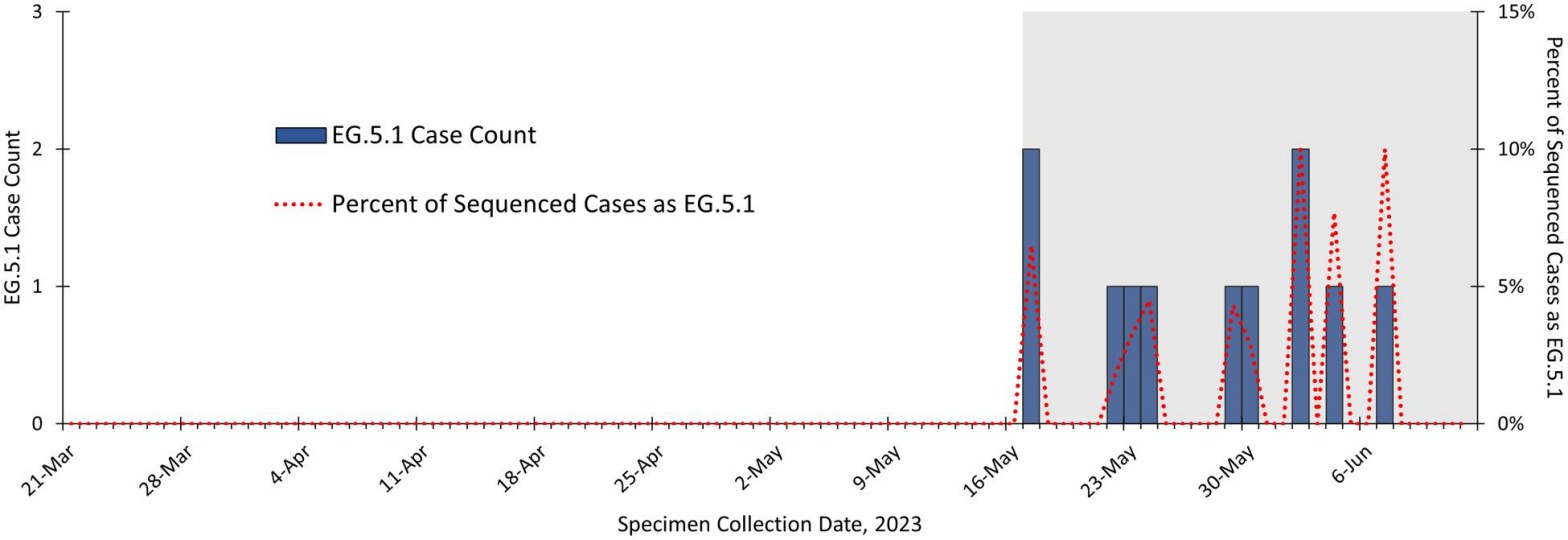
Count and percentage of cases with EG.5.1 as the SARS-CoV-2 sequencing result among NYC residents with specimens collected during 21 March–12 June 2023. Results are as of 22 June 2023, the first analysis week that EG.5.1 signaled as emerging, with the cluster window starting on 17 May 2023 shaded in gray.

The WHO designated EG.5 as a variant under monitoring on 19 July 2023 and a variant of interest on 9 August 2023 [43], 4 and 7 weeks, respectively, after our first EG.5.1 signal. During a period with many co-circulating variants and when EG.5 initially constituted a small number and percentage of cases with WGS results, the TreeScan analysis, trend visualization (Figure 3), and dendrogram (Supplementary Figure S1; see online supplementary material for a color version of this figure) led NYC Health Department officials to focus attention on this variant.

#### Delayed detection of locally emerging variants

In the analysis that was performed on 20 October 2022, multiple BE.1.1.1 subvariants signaled for the first time, indicating delayed detection of BQ.1, BQ.1.1 (RI = 19 231 years for both nodes), and BQ.1.3 (RI = 2.4 years). In the concurrent UShER version update, a subset of cases had been reassigned to BQ lineages, revealing that BQ lineages, which descended from BE.1.1.1 [40], had been present for >1 month. Once the input data were updated, TreeScan analyses appropriately detected the emergence of BQ lineages.

#### Assurance of no other locally emerging variants

The Omicron variant was first detected in NYC in clinical and wastewater samples that were collected in November 2021 and quickly became predominant [31, 44]. While staff were urgently focused on the characterization of local effects on population health, TreeScan analyses provided confirmation that additional lineages that required attention and response were not emerging concurrently.

##### Salmonella

During the first year of analyses, on 128 serotypes, TreeScan detected 16 unique clusters in the primary analysis by using the specimen collection date as the temporal element and one additional cluster in the sensitivity analysis by using the upload date (Table 5). TreeScan detects statistical anomalies, which must be investigated to distinguish true clusters from data-quality issues. Of the 17 clusters, two clusters were due to >1 isolate being sequenced from the same patient. These data-quality issues were identified by reviewing line lists and quickly resolved in SEDRIC. The remaining 15 clusters were plausibly outbreaks and worth epidemiological investigation. Of these 15 clusters, two were typhoidal and associated with travel to an endemic area. Of the 13 non-typhoidal clusters, two comprised family members with shared exposures, two reflected larger, interjurisdictional outbreaks, two were persistent strains that caused illnesses over a long time, and seven were unsolved. Of the 13 non-typhoidal clusters, one was detected at the third allele code digit so encompassed a broader allele range than rule-based cluster definitions. The remaining 12 clusters were detected to at least the fifth allele code digit (i.e. had a maximum expected difference of 4 alleles; Table 3), aligning with rule-based cluster definitions (Supplementary Appendix S2). Of these clusters, nine were concurrently detected by PHL, two consisted entirely of isolates that were tested at other jurisdictions’ public health laboratories and so could not have been detected by PHL, and one was not concurrently detected by PHL because temporary technological issues disrupted portions of PHL’s cluster detection workflow.

**Table 5.** Salmonellosis clusters among NYC residents that were detected by using prospective tree-temporal scan statistics in weekly analyses conducted during 16 November 2022–8 November 2023

Serotype	Allele code	Date first detected(DD/MM/YY)	Cluster start date (specimen collection date) (DD/MM/YY)	Observed cases	Relative risk	Excess cases	Recurrence interval	Notes
Typhi	6788.1.1.47.1.97	15/3/23	15/2/23	4	∞	4.0	481 years	Same family. All traveled to endemic area
Typhimurium	6766.47.1.12.4.3	1/3/23	10/2/23	2	∞	2.0	33 years	Unsolved
Paratyphi B Var. L(+) Tartrate+	316.6.3.13.1	19/7/23	4/7/23	2	∞	2.0	31 years	Unsolved
Typhimurium	6745.20.1.1.10.9	1/2/23	9/1/23	3	∞	3.0	12 years	Same family. Multiple shared exposures
Typhimurium	6745.56.1.2.3.116	8/12/22	25/10/22	4	∞	4.0	7.5 years	Unsolved
Newport	6809.9.1.1.1.954	12/10/23	24/9/23	2	∞	2.0	6.2 years	Persisting enteric bacterial strain
I 4:i:-	772.1.3.1.2.2	11/1/23	8/12/22	3	∞	3.0	2.9 years	Unsolved
Hadar	6771.1.1.30.1	8/11/23	17/10/23	3	126.1	3.0	2.6 years	Persisting enteric bacterial strain
Kottbus	6185.1.1.2.1	4/1/23	1/12/22	5	25.6	4.8	1.9 years	Unsolved
Typhimurium	6766.32.1.3.8.3	17/5/23	28/4/23	2	∞	2.0	339 days	Unsolved
Javiana	6765.1.1	7/9/23	21/7/23	7	14.6	6.5	213 days	Three subgroups of more closely related isolates, two of which were multistate investigations
Typhimurium	459.3.1.8.1.1	7/6/23	15/5/23	2	∞	2.0	174 days	Same family. Shared meal
Typhi	6788.1.1.4.18	3/5/23	10/4/23	2	∞	2.0	147 days	Data-quality issue: removed isolate from a follow-up specimen
Oranienburg	6760.67.167.5.1.1	21/12/22	19/11/22	2	∞	2.0	130 days	Data-quality issue: removed duplicate isolate
Paratyphi A	1082.1.3.1.1.11	19/4/23	24/3/23	2	∞	2.0	119 days	Same family. All traveled to endemic area
IIIb 61:k:1,5	135.5.33.6.1.1	27/9/23	26/9/23^a^	2	∞	2.0	114 days	Unsolved
Infantis	6747.16.3.109.1	13/9/23	28/7/23	4	∞	4.0	101 days	Multistate investigation

a This was the only unique cluster detected in sensitivity analyses in which the temporal element was the upload date instead of the specimen collection date.

Investigators considered TreeScan results to be helpful in focusing staff attention and investigation resources. For example, TreeScan clusters in NYC occasionally reflected concurrent, aberrant clusters across other jurisdictions, prompting multijurisdictional collaboration to identify common exposures. The TreeScan cluster at the third allele code digit alerted investigators to a local increase in *Salmonella* Javiana, spurring further investigation into possible subclusters. Moreover, in analysing isolates according to the location of residence, TreeScan detected clusters among NYC residents that otherwise might not have been detected because patients were tested by different laboratories. TreeScan also provided coverage when external technological issues disrupted certain processes at PHL.

## Discussion

By applying tree-temporal scan statistics prospectively to genomic surveillance data with a standardized hierarchical nomenclature, we automatically sifted through large quantities of data in minutes and generated weekly ranked shortlists of nodes with statistically unusual numbers of recent cases. This method flexibly evaluates all candidate clusters, across many degrees of genetic relatedness and date ranges. It dynamically accounts for any purely temporal trends, such as data lags or changes in WGS result availability, and minimizes false signals by adjusting for the multiplicity of nodes and cluster windows scanned. With real-time application to SARS-CoV-2 and *Salmonella* data, the NYC Health Department detected credible clusters for investigation and data-quality problems for correction.

### Limitations

WGS results were available for only 7% of COVID-19 cases and 62% of salmonellosis cases. After the federal COVID-19 public health emergency declaration ended in May 2023 and with reduced funding, specimen and sequence availability declined [45], which could have reduced population representativeness and delayed the detection of new variants. Additionally, although the NYC Health Code requires laboratories to reflexively culture certain enteric pathogens, including *Salmonella* [36], the widespread use of culture-independent diagnostic testing has reduced the proportion of salmonellosis cases with recovered isolates. Patients without WGS results cannot contribute their exposure histories to cluster investigations, making it more challenging to solve outbreaks. Improving population-based WGS data completeness, representativeness, and timeliness requires strengthening partnerships with clinics and submitting laboratories, as well as deepening investments in laboratory capacity and bioinformatics infrastructure, including applying culture-independent sequencing methods [46, 47].

Where WGS results were available, the tree nomenclature imposed certain limitations. For SARS-CoV-2, assigning Pango lineages by using UShER allowed accurate and stable lineage assignments at the expense of timeliness. As in the BQ lineages example, delays in updating nomenclature to recognize new lineage designations resulted in delayed detection. For *Salmonella*, the signal-to-noise ratio to detect a new cluster was poor for allele codes that ended in ‘x’, as the underlying four-, five-, and six-digit codes were masked due to low genomic diversity that increased the within-code distance beyond the assignment thresholds. More broadly, we rely on a standardized nomenclature, with no consideration of genetic distances between tree nodes.

TreeScan should complement, not replace, other cluster detection approaches using laboratory-based data, such as by examining allele ranges [11, 14]. Tree-temporal scan statistics could miss outbreaks where genetic or temporal clustering is weak. Zoonotic disease outbreaks, such as those associated with exposure to reptiles or backyard poultry, often involve multiple serotypes with large allelic diversity [15]. Patients’ isolates might be weakly clustered temporally for outbreaks due to persistent environmental contamination or following delays in accessing medical care or obtaining WGS results. Supplementary Appendix S3 provides additional minor limitations.

## Conclusions

By decreasing the reliance on time-consuming, manual laboratory data review and by simultaneously analysing data not only by genetic relatedness, but also by temporal clustering, TreeScan analyses can help officials to focus limited investigative resources on emerging clusters and variants. Future work could apply this approach to additional pathogens [11, 48], use additional hierarchical nomenclature systems, analyse additional pathogen characteristics (e.g. antimicrobial resistance patterns), and analyse state- and national-level data to support multijurisdictional outbreak response. Incorporating TreeScan as a free and open-source tool into analytical pipelines [49] could strengthen strategic frameworks for genomic surveillance [50], including in low- and middle-income countries.

Health departments should routinely apply multiple cluster detection methods to quickly detect different types of outbreaks. In NYC, spatiotemporal analyses have quickly detected outbreaks with strong geographic clustering before laboratory subtyping results became available. However, these methods could miss geographically diffuse outbreaks, such as those following exposure to a widely disseminated source, or outbreaks that affect only a few patients. Despite lags in subtyping data availability, such outbreaks could be detected more quickly by the application of tree-temporal scan statistics to WGS data. TreeScan thus fills an important gap in the public health practitioner’s automated cluster detection and monitoring toolkit.

## Ethics approval

The Institutional Review Board of the NYC Health Department determined this activity (No. 21–072) meets the definition of public health surveillance as set forth under 45 CFR§46.102(l)(2).

## Supplementary Material

dyaf032_Supplementary_Data

## Data Availability

SARS-CoV-2 variant data for NYC residents are available on GitHub (https://github.com/nychealth/coronavirus-data/tree/master/variants). Allele codes for *Salmonella* isolates are available to CDC partners via SEDRIC (https://www.cdc.gov/foodborne-outbreaks/php/foodsafety/tools/). SAS code for generating TreeScan input files is available on GitHub (https://github.com/CityOfNewYork/communicable-disease-surveillance-nycdohmh). The TreeScan software (www.treescan.org) and source code (https://github.com/scanstatistics/treescan) are freely available.
